# Systemic Vascular Risk Factors for Multiple Retinal Nerve Fiber Layer Defects

**DOI:** 10.1038/s41598-018-26160-7

**Published:** 2018-05-17

**Authors:** Kyoung In Jung, Seon Joo Kim, Chan Kee Park

**Affiliations:** Department of Ophthalmology and Visual Science, Seoul St. Mary’s Hospital, College of Medicine, The Catholic University of Korea, Seoul, Korea

## Abstract

Multiple retinal nerve fiber layer (RNFL) defects develop uncommonly, even though glaucomatous RNFL loss is typically observed as one RNFL defect in each quadrant. We investigated the risk factors associated with multiple RNFL defects to increase our understanding of the nature and pathogenesis of various RNFL defect patterns. Data from subjects with multiple RNFL defects (28 patients) and glaucoma patients without multiple RNFL defects (194 patients) were analyzed. The term “multiple RNFL defects” refers to three or more isolated defects separated by a comparatively normal area. Patients with multiple RNFL defects showed a higher prevalence of hypertension, end-stage renal disease, and cerebrovascular disease than those without multiple RNFL defects, both before and after propensity score matching for age and mean deviation (all *P* < 0.05). The number of patients with parafoveal visual field points depressed <5% on pattern deviation plots was higher in subjects with multiple RNFL defects than in those without multiple RNFL defects (*P* = 0.048). In conclusion, the presence of multiple RNFL defects had clinical relevance for systemic vascular risk factors and a higher risk of parafoveal scotoma. Clinicians should be aware of the possibility of concomitant systemic vascular disease when evaluating patients with multiple RNFL defects.

## Introduction

Focal retinal nerve fiber layer (RNFL) defects are rarely detected in normal eyes and are highly specific for optic nerve damage^1^. Localized RNFL defects are useful as a clinical sign of early glaucoma^1,2^. They may also indicate a substantial loss of retinal ganglion cells, as has been found in glaucoma patients with localized RNFL loss apparent on stereophotographs^3^.

In glaucomatous optic neuropathy, RNFL loss is typically observed as one RNFL defect in each sector. Uncommonly, multiple RNFL defects develop in one quadrant. They are divided by a normal RNFL within a single sector^4,5^. We speculated that multiple RNFL defects were related to systemic vascular risk factors for the following reasons. At first, multiple RNFL defects are not limited to the superior or inferior sectors of the optic disc, and many are also located at the temporal region of the optic disc^5^. In glaucoma patients, localized RNFL defects are generally detected in the inferotemporal region, followed by the superotemporal region^3^. RNFL defects adjacent to the center of the macula or paracentral visual field defects are associated with systemic vascular risk factors, rather than high intraocular pressure (IOP) in glaucoma^6–8^. Secondly, Xu *et al*. found that localized RNFL defects were associated with arterial hypertension^9^. In that study, the authors presented one case with arterial hypertension and multiple localized RNFL defects located at 10, 11, and 12 o’clock^9^. Given those findings, it is possible that multiple RNFL defects are related to systemic vascular factors. Another previous study reported that ocular factors, such as myopia and a small optic disc, were associated with multiple RNFL defects in glaucoma^5^. However, there has been no study directly analyzing the influence of systemic vascular disease on the development of multiple RNFL defects.

In this study, we evaluated whether systemic vascular risk factors are related to multiple RNFL defects. The characteristics of visual field defects in patients with multiple RNFL defects were also assessed. This investigation will increase our understanding of the nature and pathogenesis of various RNFL loss patterns.

## Methods

The Institutional Review Board of the Catholic University of Korea, Seoul, Korea, approved this cross-sectional study and waived the need for written informed consent because of its retrospective design. This study followed the tenets of the Declaration of Helsinki. Inclusion criteria were a best-corrected visual acuity of 20/40 or better, spherical equivalent >−7 diopters (D), and a normal open angle. Patients with trauma or uveitis or proliferative diabetic retinopathy or rim pallor or diseases that might affect the peripapillary area were excluded. Patients with abnormal color vision were excluded. All patients underwent complete ophthalmic examination, including slit-lamp biomicroscopy, Goldmann applanation tonometry, gonioscopy, central corneal thickness measurement, and dilated fundus biomicroscopy. All subjects underwent stereoscopic optic disc imaging. Color vision testing was performed using the 24-plate edition of the Ishihara test book published in 1997 (Kanehara & Co., Ltd, Tokyo, Japan). The spherical equivalent was calculated as the spherical error +1/2 cylinders. Myopia was defined as a spherical equivalent ≤−1 diopter. Systemic risk factors, including diabetes, hypertension, end-stage renal disease, heart disease, cerebrovascular disease, and hematologic disease, were analyzed. The systemic diseases were divided into common and uncommon diseases. The cut-off value for the prevalence of common diseases was 10.0% in total subjects.

Multiple RNFL defects were defined as 3 or more RNFL defects separated by relatively normal RNFL regions^5^. Multiple RNFL defects were composed of only focal RNFL defects or those mixed with diffuse defects. Focal RNFL defects were defined as a dark gray slit or wedge without sliver striations. The width of the focal defect was wider than a major retinal vessel at a one-disc diameter length from the margin of the optic disc. Patients with multiple RNFL defects who met the inclusion criteria were consecutively included from all patients examined for glaucoma at the glaucoma clinic of Seoul St. Mary’s Hospital between May 2012 and May 2016. When both eyes met the inclusion criteria, one eye per individual was randomly selected for the study.

**Figure 1:**
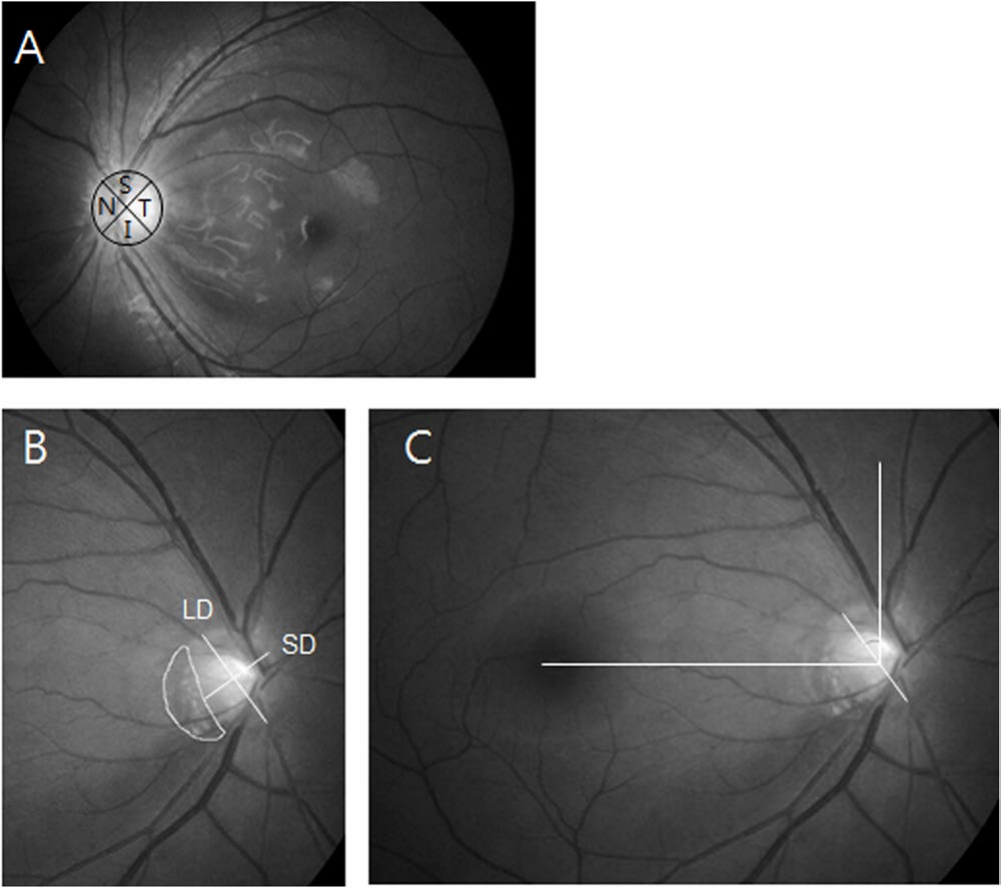
Demonstration of quadrant locations of split retinal nerve fiber layer (RNFL) defects and posterior pole profiles. (**A**) The location of split RNFL defects was determined by quadrant (superior, temporal, inferior, nasal, equal to 90 degrees) around the optic disc on red-free imaging. (**B**) Tilt ratio was identified as the ratio between the longest diameter (LD) and the shortest diameter (SD) of the optic disc. The β-zone parapapillary atrophy (PPA) area was calculated using the Image J program. (**C**) Torsion degree was calculated as the angle between the LD from the vertical meridian, which was at right angles to the reference line. (**A**) I, inferior; N, nasal; S, superior; T, temporal (**B**) LD, longest diameter; SD; shortest diameter.

Data for preperimetric or perimetric glaucoma patients were collected from all patients examined for glaucoma at the glaucoma clinic of Seoul St. Mary’s Hospital between September and December 2015. This study included eyes with the presence of a glaucomatous optic disc, such as those with diffuse or focal rim thinning, notching, or retinal nerve fiber layer defects with or without corresponding glaucomatous visual field (VF) defects. Subjects with and without multiple RNFL defects were compared based on demographics including systemic risk factors, RNFL and optic disc parameters, posterior pole profiles, and the characteristics of visual field defects. The location of split RNFL defects was indicated as a quadrant (superior, temporal, inferior, nasal, each 90 degrees) around the optic disc on red-free photography (Fig. 1).

### Optical Coherence Tomography

Spectral-domain optical coherence tomography (SD-OCT) imaging was performed using Cirrus HD-OCT version 6.0 (Carl Zeiss Meditec, Inc.). RNFL thickness was measured using optic Disc Cube 200 × 200 scan mode. The Cirrus SD-OCT automatically determines the center of the disc and then draws a circumpapillary circle (radius: 1.73 mm) from the cube data set for RNFL thickness analysis. Poor quality images with signal strength less than 6 were discarded. Mean average RNFL thickness, or the mean thickness in each of 4 quadrants, was provided by the software and included in the comparison between the two groups according to multiple RNFL defects. Optic disc parameters such as disc area, rim area, average cup-to-disc ratio (CDR), vertical CDR, and cup volume were analyzed.

### Posterior Pole Profiles

The red-free RNFL images were measured to evaluate the tilt ratio, optic disc torsion, and ratio of βPPA area to disc area. Two examiners (K.I.J., K.S.J.) blinded to clinical information measured the images using the Image J 1.48 program (https://imagej.nih.gov/ij/download.html, National Institutes of Health, Bethesda, MD). The means of the 2 measurements of the 2 examiners were included in this study (Fig. 1).

Tilt ratio (shortest diameter/shortest diameter) was employed to express optic disc tilt^10,11^. Optic disc torsion was determined as the deviation of the long optic disc axis from the vertical meridian, which was at right angles to a reference line coupling the fovea and disc center. The absolute value of optic disc torsion was used in the analysis^12,13^. β-zone parapapillary atrophy (PPA) was indicated as an inner crescent of chorioretinal atrophy with observable sclera and choroidal vessels. The disc and βPPA border were outlined using a measurement tool in Image J software, which assessed the areas of the disc and βPPA and displayed them in a separate window. The ratio of βPPA area to disc area was used.

### Visual Field Testing

All subjects underwent standard automated perimetry using 24-2 Swedish interactive threshold algorithm (SITA) standard programs with a Humphrey field analyzer II 750i (Carl Zeiss Meditec, Dublin, CA). Mean deviation (MD) and PSD were used in the analysis. A glaucomatous VF defect was defined as a cluster of 3 or more points with a *P* value <5%, one of which had a *P* value of <1% on the pattern deviation plot. Reliable tests were defined as < 15% fixation losses, false positives, or false negatives.

On four central 10° VF points of pattern deviation plot, the presence or percentages of significantly depressed points at P < 0.05 were evaluated. On 12 central VF points of pattern deviation plot, each pattern deviation value was compared between subjects with and without multiple RNFL defects.

### Statistical Analysis

Differences between the different groups were evaluated by Student’s *t* test or chi-squared test. Multiple logistic regression analysis was performed to determine which systemic vascular disease was associated with multiple RNFL defects. To decrease the chance of selection bias, propensity score analysis matching was performed. This analysis can perform a quasi-randomized comparison from a retrospective study^14^. To determine the propensity score, multiple logistic regression analysis was performed using age and MD with SPSS software (ver. 22.0; SPSS Inc., Chicago, IL)^15^. The propensity score was defined for each participant as the probability (from 0 to 1) of assignment to the case group, given covariates such as age and MD. A logistic regression model is estimated in which an indicator variable denoting the group status (Z, where Z equals 1 if the subject is in the case group or 0 if the subject is in the control group) is regressed according to covariates (independent variables, xi: age, MD in this study). A 1:3 matching method was chosen with the nearest neighbor approach without replacement, based on a greedy matching method. A 1:3 matching means that 3 control units with similar propensity scores were matched to a single unit in the case group. Eighty-four patients, three times the number of subjects with multiple RNFL defects (n = 28), were selected from the group of 194 glaucoma patients without multiple RNFL defects. One-to-many nearest neighbor matching can provide higher precision with little cost in bias compared with simple 1:1 matching^16^. The technical details are described as follows. We first installed the R package (R 2.15.0), the program to connect R and SPSS (essentials for R), and the PS matching program. The PS matching program was written as a custom dialog in SPSS. This program operates in R through the SPSS-R-plugin. *P* < 0.05 indicated statistical significance.

## Results

Data from 28 patients with multiple RNFL defects and 194 patients with glaucoma were analyzed. Twenty-six (92.9%) of 28 patients with multiple RNFL defects exhibited abnormalities in the thickness map obtained with SD-OCT. After 1:3 propensity score matching, 84 patients with glaucoma were selected from among 194 patients with glaucoma. Patients without multiple RNFL defects (average age 57.0 ± 13.3 years) were older than those with multiple RNFL defects (average age 52.7 ± 11.4 years), but the difference was not statistically significant (*P* = 0.103; Table 1). MD in the visual field showed more severe glaucomatous damage in subjects without multiple RNFL defects (−6.7 ± 7.3 dB) than in those with multiple RNFL defects (−2.8 ± 3.2 dB; *P* < 0.001). After propensity score matching according to age and MD, the two groups demonstrated similar glaucomatous stage by MD (*P* = 0.995). There were no significant differences in best corrected visual acuity, spherical equivalent, untreated IOP, or occurrence of disc hemorrhage between the two groups before and after propensity score matching. In terms of systemic factors, the proportion of patients with hypertension was higher in patients with multiple RNFL defects than those without them among common vascular diseases, both before and after propensity score matching (all *P* < 0.01) Among uncommon diseases, the proportion of patients with end-stage renal disease, and cerebrovascular disease was higher in patients with multiple RNFL defects than in those without them, both before and after propensity score matching (all *P* < 0.01).

**Table 1:** Demographics of patients

Parameter			Before propensity score matching			After propensity score matching		
			Multiple RNFL defect (+) (n = 28)	Multiple RNFL defect (−) (n = 194)	*P* value	Multiple RNFL defect (+) (n = 28)	Multiple RNFL defect (−)(n = 84)	*P* value
Age (years)			52.7 ± 11.4	57.0 ± 13.3	0.103	52.7 ± 11.4	53.3 ± 11.7	0.808
Percentage of female (%)			14(50.0%)	119(61.3%)	0.304	14(50.0%)	52(61.9%)	0.278
Systemic factors	Common diseases	Diabetes(%)	5(17.9%)	21(10.9%)	0.341	5(17.9%)	5(6.0%)	0.117
		Hypertension (%)	17(60.7%)	33(17.1%)	**<0.001**	17(60.7%)	10(11.9%)	**<0.001**
	Uncommon diseases	End-stage renal disease(%)	7(25.0%)	1(0.5%)	**<0.001**	7(25.0%)	0(0.0%)	**<0.001**
		Heart disease(%)	2(7.1%)	7(3.6%)	0.317	2(7.1%)	2(2.4%)	0.260
		Cerebrovascular disease(%)	5(17.9%)	2(1.0%)	**<0.001**	5(17.9%)	1(1.2%)	**0.004**
		Hematologic disease(%)	2(7.1%)	0(0.0%)	**<0.001**	2(7.1%)	0(0.0%)	0.061
Ocular factors	Best corrected visual acuity(logMAR)		0.03 ± 0.05	0.05 ± 0.12	0.081	0.03 ± 0.05	0.02 ± 0.11	0.667
	Central corneal thickness (µm)		536.3 ± 35.6	534.0 ± 34.0	0.754	536.3 ± 35.6	535.3 ± 36.3	0.902
	Spherical equivalent (diopter)		−2.2 ± 3.0	−1.4 ± 2.3	0.145	−2.2 ± 3.0	−2.0 ± 2.5	0.839
	Myopia (%)		16(57.1%)	100(51.5%)	0.579	16(57.1%)	52(61.9%)	0.655
	Untreated intraocular pressure (mmHg)		15.1 ± 5.1	17.2 ± 8.5	0.078	15.1 ± 5.1	16.0 ± 7.0	0.530
	Mean deviation (dB)		−2.8 ± 3.2	−6.7 ± 7.3	**<0.001**	−2.8 ± 3.2	−2.8 ± 2.9	0.995
	Pattern standard deviation (dB)		3.9 ± 3.2	6.3 ± 4.5	**0.001**	3.9 ± 3.2	4.1 ± 3.5	0.758
	Disc hemorrhage (%)		2(7.1%)	30(15.5%)	0.387	2(7.1%)	18(21.4%)	0.151

RNFL, retinal nerve fiber layer.

Continuous variables are expressed as n (percentage) or mean ± standard deviation.

^*^Statistically significant differences between two groups (*P* < 0.05) by Student’s t-test for continuous variables or chi-squared test for categorical data are indicated in bold.

Table 2 shows optic disc characteristics and RNFL thickness parameters according to the presence of multiple RNFL defects. Average or quadrant RNFL thickness did not show a difference between patients with multiple RNFL defects and those without after propensity score matching. Disc area did not differ depending on the presence of multiple RNFL defects both before (*P* = 0.162) and after (*P* = 0.142) propensity score matching. Rim area, average CDR, vertical CDR, and cup volume showed more glaucomatous damage in subjects without multiple RNFL defects compared to those with multiple RNFL defects. Patients with multiple RNFL defects (+) presented with a smaller degree of disc torsion and smaller PPA area to disc area ratio than the multiple RNFL defect (−) group both before (Both *P* < 0.001) and after (P = 0.012, 0.018, respectively) propensity score matching.

**Table 2:** Optic disc and retinal never fiber layer parameters, and posterior pole profiles.

Parameter		Before propensity score matching			After propensity score matching		
		**Multiple RNFL defect (+)** (n = 28)	**Multiple RNFL defect (−)** (n = 194)	*P* value	**Multiple RNFL defect (+)** (n = 28)	**Multiple RNFL defect (−)** (n = 84)	*P* value
Optic disc parameters (Cirrus OCT)	Disc area (mm^2^)	1.89 ± 0.53	2.04 ± 0.40	0.162	1.89 ± 0.53	2.04 ± 0.42	0.142
	Rim area (mm^2^)	1.06 ± 0.31	0.77 ± 0.20	**<0.001**	1.06 ± 0.31	0.83 ± 0.17	**0.001**
	Average CDR	0.63 ± 0.19	0.77 ± 0.09	**0.001**	0.63 ± 0.19	0.75 ± 0.09	**<0.001**
	Vertical CDR	0.64 ± 0.19	0.77 ± 0.10	**0.002**	0.64 ± 0.19	0.74 ± 0.10	**0.011**
	Cup volume (mm^3^)	0.33 ± 0.24	0.56 ± 0.29	**<0.001**	0.33 ± 0.24	0.54 ± 0.28	**0.001**
Posterior pole profiles	Tilt ratio	1.1 ± 0.1	1.2 ± 0.9	0.788	1.1 ± 0.1	1.1 ± 0.2	0.458
	Disc torsion (degree)	8.4 ± 3.9	14.1 ± 8.2	**<0.001**	8.4 ± 3.9	11.6 ± 6.2	**0.012**
	PPA area/disc area ratio	0.3 ± 0.2	0.4 ± 0.3	**<0.001**	0.3 ± 0.2	0.4 ± 0.2	**0.018**
Retinal nerve fiber layer thickness (µm)	Average	77.0 ± 11.5	72.5 ± 13.1	0.096	77.0 ± 11.5	76.2 ± 11.5	0.767
	Superior	99.3 ± 20.3	90.0 ± 21.5	**0.035**	99.3 ± 20.3	95.0 ± 18.5	0.317
	Nasal	60.2 ± 8.6	60.9 ± 10.3	0.735	60.2 ± 8.6	61.4 ± 10.9	0.583
	Inferior	88.9 ± 21.3	78.8 ± 21.0	**0.021**	88.9 ± 21.3	84.9 ± 20.3	0.377
	Temporal	58.2 ± 20.0	60.0 ± 12.9	0.546	58.2 ± 20.0	62.8 ± 12.8	0.165

CDR, cup-to-disc ratio; OCT, optical coherence tomography; PPA, parapapillary atrophy.

^*^Statistically significant differences between two groups (*P* < 0.05) by Student’s t-test are indicated in bold.

**Figure 2:**
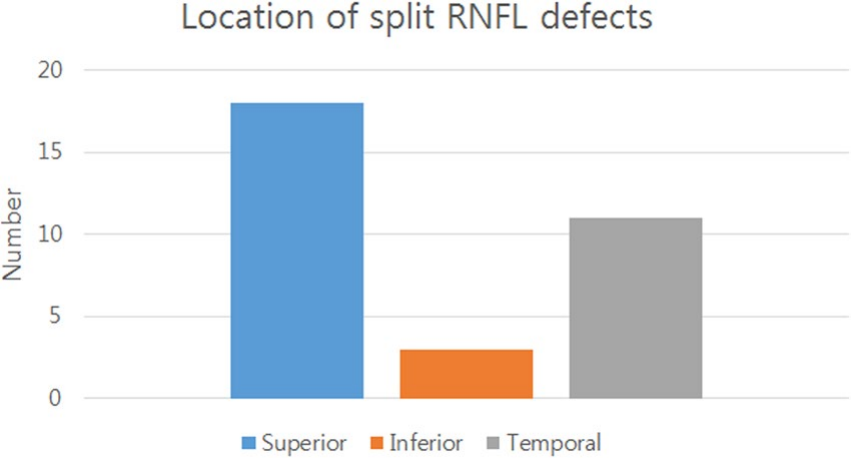
Split retinal nerve fiber layer (RNFL) defects developed most frequently in the superior region of the optic disc (n = 18), followed by the temporal region (n = 11). Split RNFL defects (n = 3) occurred less frequently in the inferior sector of the optic disc.

Many of the split RNFL defects were in the superior quadrant of the optic disc (n = 18), followed by the temporal (n = 11) and inferior (n = 3) regions of the optic disc (Fig. 2).

Table 3 presents the involvement of the paracentral visual field defect within the 4 central points. The proportion of patients with paracentral visual field points depressed <5% on pattern deviation plot was higher in the multiple RNFL defect (+) group than in the multiple RNFL defect (−) group (*P* = 0.048). Among these patients, 10 (58.8%) had an isolated parafoveal scotoma, and 7 (41.2%) had combined parafoveal scotomas. Other visual field defect patterns were found as follows: Three patients had nasal scotoma. Five patients had visual field defects in the Bjerrum area. Three patients had no visual field defect.

**Table 3:** Paracentral visual field points depressed <5% on pattern deviation plot.

Pattern deviation plot		**Multiple RNFL defect (+)** (n = 28)	**Multiple RNFL defect (−)** (n = 84)	*P* value^*^
Points depressed <5%	Yes	17 (60.7%)	11(39.3%)	**0.048**
	Mean number	1.0 ± 1.2	0.6 ± 0.9	0.075

^*^Statistically significant differences between two groups (*P* < 0.05) by Student’s t-test for continuous variables or chi-squared test for categorical data are indicated in bold.

On a pattern deviation plot, 12 paracentral visual field points were compared between the two groups. In subjects with multiple RNFL defects, the pattern deviation value (−2.3 dB) of the lower paracentral visual field points was lower than in those without them (−1.1 dB, *P* = 0.015; Supplementary Fig. S1). Pattern deviation (−6.6 dB) of the upper paracentral VF points displayed a lower value in patients without multiple RNFL defects than in patients with multiple RNFL defects (−3.2 dB, P = 0.004).

Among common vascular diseases, hypertension was associated with multiple RNFL defects by multiple logistic regression analysis (*P* < 0.001; Table 4). Among uncommon diseases, end-stage renal disease and cerebrovascular disease were both related to multiple RNFL defects by multiple logistic regression analysis (both *P* < 0.001; Table 4)

**Table 4:** Multiple logistic analysis of systemic factors associated with multiple retinal nerve fiber layer defect.

		Odds ratio	95% CI		*P* value
Common diseases	Diabetes				0.991
	Hypertension	7.49	1.96	17.45	**<0.001**
Uncommon diseases	End-stage renal disease	73.70	8.30	654.47	**<0.001**
	Heart disease				0.482
	Cerebrovascular disease	26.60	4.49	157.53	**<0.001**
	Hematologic disease				0.999

^*^Statistically significant differences (*P* < 0.05) assessed by logistic regression analysis are indicated in bold.

**Figure 3:**
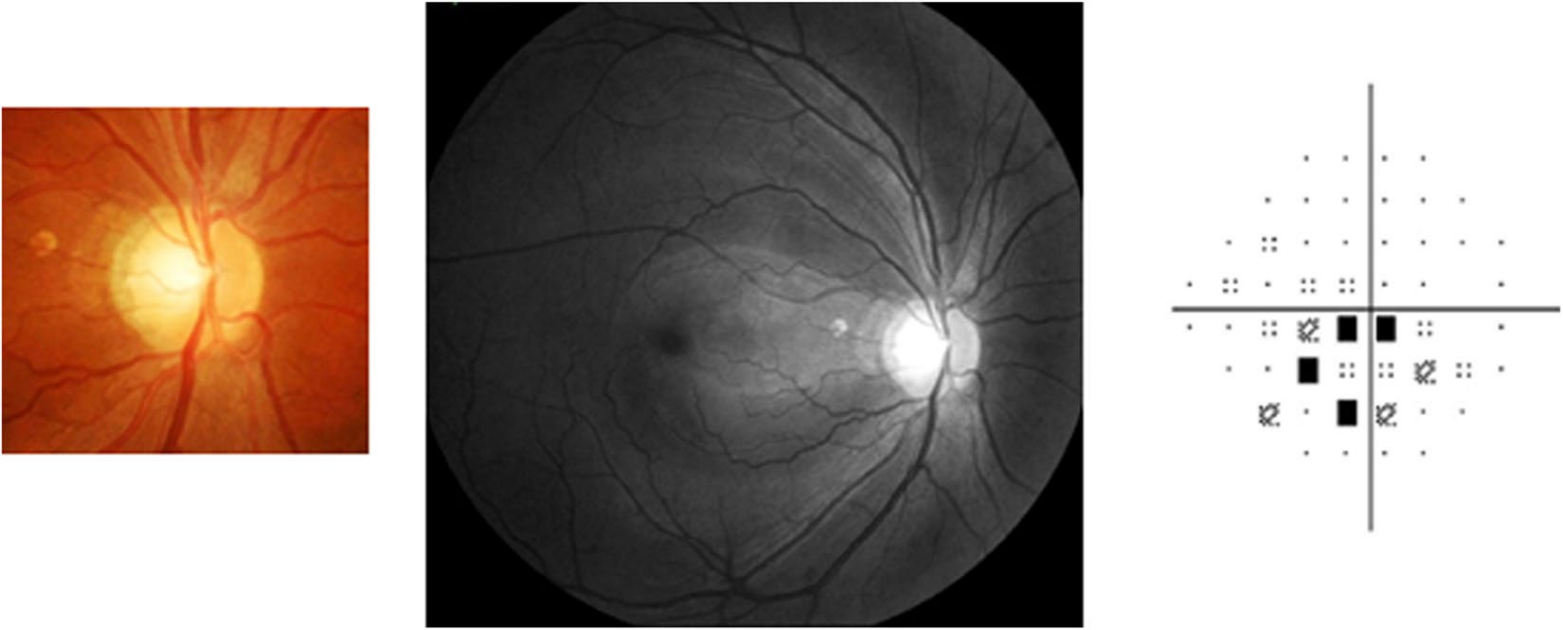
A Representative case with multiple retinal nerve fiber layer (RNFL) defects. A 47-year-old man with a history of systemic hypertension showed multiple RNFL defects in his left eye. His visual field damage also involved paracentral defect.

A representative case with multiple RNFL defects is shown in Fig. 3. A 47-year-old male with a history of systemic hypertension showed multiple RNFL defects in his left eye. The best-corrected visual acuity in their left eyes was 20/20, and no abnormal color vision was identified.

**Figure 4:**
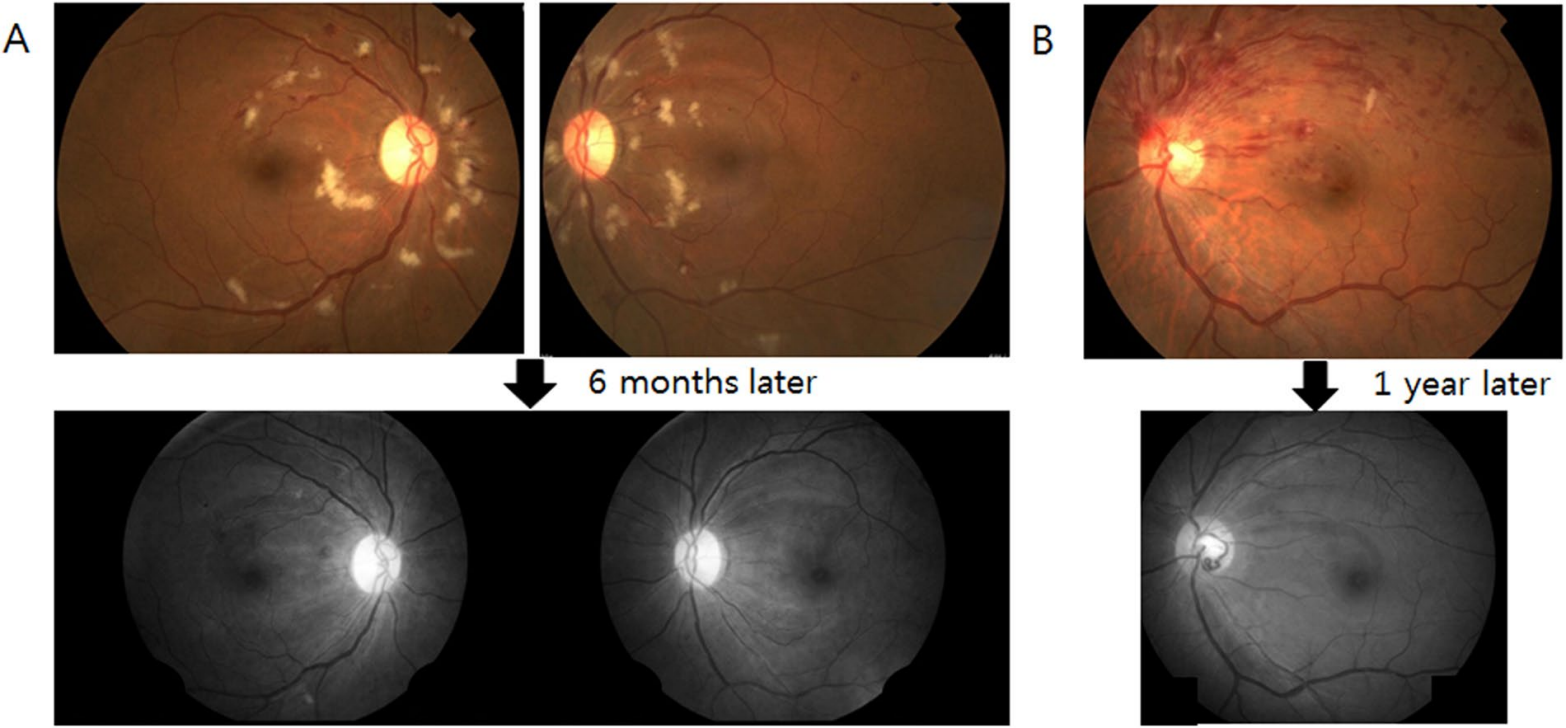
Two cases with multiple retinal nerve fiber layer (RNFL) defects related to retinal vascular disease. (**A**) A 43-year-old female with moderate non-proliferative diabetic retinopathy, hypertensive retinopathy, and a history of cerebral infarction presented with multiple cotton-wool spots in both eyes. Six months later, multiple RNFL defects occurred in both eyes. (**B**) A 42-year-old female with systemic hypertension showed multiple RNFL defects at the sector of the optic disc corresponding to the location where a branched retinal vein obstruction had occurred.

Among subjects with multiple RNFL defects, only two cases were associated with retinal vascular disease. A 43-year-old female with moderate diabetic retinopathy, hypertensive retinopathy, and history of cerebral infarction showed multiple cotton-wool spots in both eyes (Fig. 4). Six months later, she was referred to the glaucoma clinic because of elevated IOP (22–23 mmHg). Her fundi showed multiple RNFL defects in both eyes. Second, a 42-year-old female with systemic hypertension developed scotoma suddenly in her left eye and visited our hospital. Her fundus imaging demonstrated branched retinal vein obstruction at the superotemporal arcade. After loss to follow-up for one year, she was referred to our glaucoma clinic from a local clinic because of atypical retinal nerve fiber layer defects. She showed multiple RNFL defects in the superotemporal sector of the optic disc corresponding to the location where branched retinal vein obstruction had occurred.

## Discussion

We demonstrated that multiple RNFL defects were associated with systemic hypertension among common systemic vascular diseases, and end-stage renal disease and cerebrovascular disease among uncommon diseases. Patients with multiple RNFL defects had higher odds for parafoveal field defects than glaucoma patients without multiple RNFL defects.

In 1992, a previous study reported that myopia and a small optic disc were related to multiple RNFL defects in glaucoma^5^. However, that study did not investigate systemic risk factors. Optic disc size was measured only via red-free fundus imaging. They did not exclude eyes with high myopia. In our study, highly myopic eyes (spherical equivalent >−7 D) were excluded because atrophic changes of the neural retina and choroid could obscure a clear pattern of RNFL defects. Posterior pole profiles, such as a tilted optic disc, greater degree of disc torsion, and β-zone PPA, were not associated with multiple RNFL defects. Posterior pole profiles are influenced by myopia^12^. At least within non-highly myopic eyes, myopia and its associated characteristics did not seem to play a critical role in the development of multiple RNFL defects.

Seventeen patients (60.7%) with multiple RNFL defects had a history of systemic hypertension. Systemic hypertension increased the risk of developing glaucoma in meta-analyses^17,18^. CDR or cup volume were smaller in patients with multiple RNFL defects than glaucoma patients without multiple RNFL defects and with similar VF defects. The pathogenesis of multiple RNFL defects in patients with systemic hypertension does not seem to be identical to that of glaucomatous optic neuropathy. One report found that localized RNFL defects were related to arterial hypertension, especially in the severe stage of hypertension^9^. The odds ratio for the connection between systemic hypertension and localized RNFL defects was higher than for the connection between systemic hypertension and retinal microvascular abnormalities^9^. In moderate hypertensive retinopathy, cotton-wool spots, which are accumulations of axoplasmic material of RNFLs, can be detected as a result of focal infarcts of the RNFL. In several case reports, a giant cotton-wool spot left a RNFL defect as a sequela to the cotton-wool spot^19,20^. In our study, only one case showed a previous history of cotton-wool spots, although we could not exclude the possibility that cotton-wool spots developed and disappeared completely in other cases. We cannot conclusively determine whether multiple RNFL defects in subjects with a history of systemic hypertension were caused by glaucoma, cotton-wool spots or a combination of both. Systemic hypertension is the major factor contributing to target organ damage involving the brain, kidney, and heart. Among uncommon vascular diseases, end-stage renal disease and cerebrovascular disease were related to systemic hypertension in five cases (71.4%) and four cases (80.0%), respectively.

In patients with multiple RNFL defects, the number of RNFL defects was greater than in those with single glaucomatous RNFL defects in one quadrant. However, multiple RNFL defects were separated by relatively normal RNFL regions. Total area of RNFL defects may be similar between the multiple RNFL defect (+) and (−) groups. Therefore, we speculate that there was no difference in average RNFL thickness between the two groups because the total areas of the RNFL defects were similar between the two groups.

Split RNFL defects appeared more often in the superior quadrant (18 cases) of the optic disc than in the inferior quadrant (3 cases), even though OCT showed thinner RNFL thicknesses in the inferior quadrant (88.9 ± 21.3 µm) than in the superior quadrant (99.3 ± 20.3 µm) in cases with multiple RNFL defects. The VF pattern deviation plot showed more significant glaucomatous defects on the inferior paracentral field in subjects with multiple RNFL defects compared to those without them (P = 0.015). In glaucoma, localized RNFL defects are usually observed in the inferotemporal region, followed by the superotemporal region^3^. Larger lamina pore size with less supporting connective tissue at the inferotemporal optic nerve head may make retinal ganglion cells in this region vulnerable to glaucomatous damage and cause initial inferotemporal RNFL defects in many cases of glaucoma^21,22^. The strong association with systemic vascular disease seemed to play a role in the predominance of split RNFL defects in the superior sector of the optic disc. Vascular insufficiency or disturbances to the optic nerve head may lead to more split RNFL defects at the superior side of the optic disc due to gravitational effects^23^. Several studies, including a previous study from our group, have found that RNFL loss in type 2 diabetic patients showed a predominance in the superior region of the optic nerve^23–25^. Multiple RNFL defects have different characteristics in the susceptible region of the optic disc compared to typical glaucomatous RNFL defects.

Damage to the papillomacular bundle or central fibers can induce central or paracentral visual field defects. Early glaucomatous damage in the macula is clinically critical because central visual disturbance puts patients at greater risk of losing visual function and interferes with everyday activities^26–28^. Accumulating clinical evidence suggests that paracentral scotoma may occur with non-pressure factors either in addition to or rather than IOP-dependent factors^6–8^. Parafoveal scotoma developed more frequently in patients with normal tension glaucoma than in patients with high tension glaucoma in several studies^29,30^, while other studies did not show consistent findings^31,32^. Our group found that glaucoma patients with autonomic dysfunction and abnormal peripheral microcirculation more often presented with paracentral visual field defects than patients without these dysfunctions^7^. Therefore, we speculated that the development of multiple RNFL defects related to systemic vascular disease might affect frequent involvement of paracentral visual field defects. However, multiple RNFL defects did not result in a distinct loss of visual acuity because they did not involve the fovea. Mean best corrected visual acuity of patients with multiple RNFL defects was 0.03 ± 0.05 (logMAR), similar to that of glaucoma patients without multiple RNFL defects.

In two cases, split RNFL defects were associated with the previous occurrence of retinal vascular disease, such as hypertensive retinopathy or branched retinal vein occlusion. Several studies have found RNFL defects or thinning after resolution of retinal vein occlusion^33,34^. However, a pattern of defects, such as multiple RNFL defects, has not been reported.

One of the limitations of this study is its retrospective nature. We sought to overcome the limitations of our study by using propensity-score matching according to age and MD in the comparison of the characteristics of eyes with multiple RNFL defects to those with typical glaucomatous damage. This study also has a possibility of selection bias due to its hospital-based nature with a small sample size. A small group of patients with multiple RNFL defects may not be representative of the larger population with them. Several subjects with multiple RNFL loss and rare diseases, such as trauma and pancreatitis, might not be included. Our research group attempted to overcome the limitations by reviewing charts over a long period of time—approximately 4 years. Hospital-based studies can provide important clues as to whether particular attributes are related to disease. Systemic hypotension has been suggested to be a risk factor for glaucoma. However, blood pressure was not measured directly in this retrospective study. We could not obtain exact information with regard to systemic hypotension due to the nature of this retrospective study. That is one of limitations of this study. Lack of detailed information on systemic hypertension such as its duration or stage, is also one of this study’s limitations. Overall, the clinical findings supporting the diagnosis of glaucoma in patients with multiple RNFL defects are more prominent. Patients with multiple RNFL defects showed definitely the presence of the RNFL defect. RNFL thickness was thinner in the inferior quadrant (88.9 ± 21.3 µm) than in the superior quadrant (99.3 ± 20.3 µm) in cases with multiple RNFL defects. There was no significant difference in the maximal IOP between the group with multiple RNFL defects and those without multiple RNFL defects (P = 0.144). We excluded patients with trauma, rim pallor, abnormal color vision and a best-corrected visual acuity of less than 20/40 to decrease the possibility of including other optic nerve diseases. In fact, no one with multiple RNFL defects was excluded because of abnormal color vision or decreased best-corrected visual acuity. Vascular insufficiency or instability has been suggested as a vascular pathogenic mechanism underlying development or progression of glaucoma. In this study, multiple RNFL defects were associated with systemic vascular risk factors. We speculated that vascular insufficiency might play a prominent role relatively than the compression of lamina cribrosa in patients with multiple RNFL defects, even though the confirmatory study is needed. Therefore, patients with multiple RNFL defects seemed to have *multiple* RNFL defects and less optic disc change than glaucoma patients without multiple RNFL defects. When glaucomatous optic neuropathy occurs predominantly with vascular insufficiency, it seemed that there might be a high possibility of developing multiple RNFL defects. Alterations in the optic disc or RNFL due to glaucomatous structural changes might induce hemodynamic changes at that location. Blood flow stasis found by angiography in glaucoma patients can result in endothelial damage or thrombosis formation^35^. Therefore, we assumed that glaucomatous damage also could contribute to formation of multiple RNFL defects because multiple RNFL defects were related to systemic vascular risk factors. However, it is not clear whether all cases with multiple RNFL defects independently develop from the pathogenesis of glaucoma or in combination with glaucoma. It was one of the limitations of our study. Larger and more carefully controlled population-based studies are needed to confirm the results of this study.

In conclusion, multiple RNFL defects were associated with systemic vascular risk factors and frequent involvement of parafoveal scotoma. Further studies are needed to determine whether nerve damage with multiple RNFL defects progresses or not, and if this occurs rapidly or slowly. Clinicians must consider the possibility of concomitant systemic vascular disease when evaluating patients with multiple RNFL defects.

## Electronic supplementary material

Supplementary Fig.1
