# Supplementary material for: Systemic Vascular Risk Factors for Multiple Retinal Nerve Fiber Layer Defects
Source: Sci Rep. 2018 May 17;8:7797. doi: 10.1038/s41598-018-26160-7 (PMC5958060; doi:10.1038/s41598-018-26160-7)
Supplement: Supplementary file 1 — Supplementary Fig.1 [file 41598_2018_26160_MOESM1_ESM.pdf]

Systemic Vascular Risk Factors for Multiple Retinal Nerve Fiber Layer Defects

Kyoung In Jung, M.D., Ph.D., Kim Seon Joo, M.D., Chan Kee Park, M.D., Ph.D.

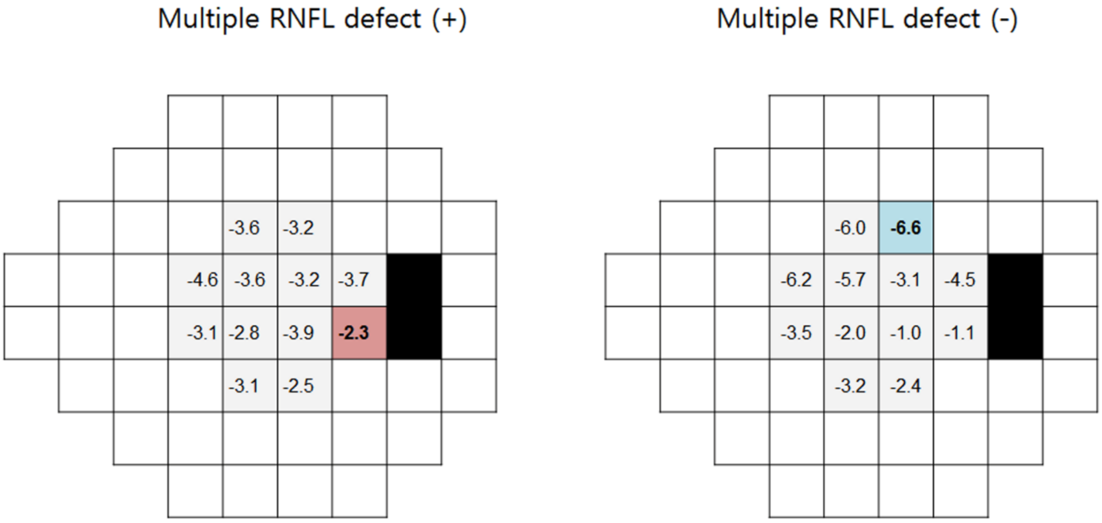

Supplementary Fig.1 In the multiple retinal nerve fiber layer (RNFL) defect (+) group, pattern deviation (-2.3 dB) of lower paracentral visual field points was lower than that of the multiple RNFL defect (-) group (-1.1 dB, P=0.015). Patients without multiple RNFL defects showed lower pattern deviation (-6.6 dB) of upper paracentral VF points than those with multiple RNFL defects (-3.2 dB, P=0.004).
